# Coating of SPIONs with a Cysteine-Decorated Copolyester: A Possible Novel Nanoplatform for Enzymatic Release

**DOI:** 10.3390/pharmaceutics15031000

**Published:** 2023-03-20

**Authors:** Jeovandro Maria Beltrame, Brena Beatriz Pereira Ribeiro, Camila Guindani, Graziâni Candiotto, Karina Bettega Felipe, Rodrigo Lucas, Alexandre D’Agostini Zottis, Eduardo Isoppo, Claudia Sayer, Pedro Henrique Hermes de Araújo

**Affiliations:** 1 Department of Chemical Engineering and Food Engineering, Federal University of Santa Catarina (EQA/UFSC), Florianópolis 88040-900, SC, Brazil; 2 Chemical Engineering Program, COPPE, Federal University of Rio de Janeiro (PEQ/COPPE/UFRJ), Rio de Janeiro 21941-972, RJ, Brazil; 3 Institute of Physics, Federal University of Rio de Janeiro, Rio de Janeiro 21941-909, RJ, Brazil; 4 Department of Clinic Analysis, Federal University of Paraná DAC/UFPR, Curitiba 80.210-170, PR, Brazil; 5 Foundation Osvaldo Cruz (FIOCRUZ), Curitiba 81350-010, PR, Brazil; 6 Academic Department of Health and Services, NANOTEC Group, Federal Institute of Santa Catarina, Florianópolis 88020-300, SC, Brazil; 7 Central Laboratory of Electron Microscopy, Federal University of Santa Catarina, LCME/UFSC, Florianópolis 88040-900, SC, Brazil

**Keywords:** copolyester, SPION, cysteine, bioconjugation, and enzymatic release

## Abstract

Superparamagnetic iron oxide nanoparticles (SPIONs) have their use approved for the diagnosis/treatment of malignant tumors and can be metabolized by the organism. To prevent embolism caused by these nanoparticles, they need to be coated with biocompatible and non-cytotoxic materials. Here, we synthesized an unsaturated and biocompatible copolyester, poly (globalide-*co*-ε-caprolactone) (PGlCL), and modified it with the amino acid cysteine (Cys) via a thiol-ene reaction (PGlCLCys). The Cys-modified copolymer presented reduced crystallinity and increased hydrophilicity in comparison to PGlCL, thus being used for the coating of SPIONS (SPION@PGlCLCys). Additionally, cysteine pendant groups at the particle’s surface allowed the direct conjugation of (bio)molecules that establish specific interactions with tumor cells (MDA-MB 231). The conjugation of either folic acid (FA) or the anti-cancer drug methotrexate (MTX) was carried out directly on the amine groups of cysteine molecules present in the SPION@PGlCLCys surface (SPION@PGlCLCys_FA and SPION@PGlCLCys_MTX) by carbodiimide-mediated coupling, leading to the formation of amide bonds, with conjugation efficiencies of 62% for FA and 60% for MTX. Then, the release of MTX from the nanoparticle surface was evaluated using a protease at 37 °C in phosphate buffer pH~5.3. It was found that 45% of MTX conjugated to the SPIONs were released after 72 h. Cell viability was measured by MTT assay, and after 72 h, 25% reduction in cell viability of tumor cells was observed. Thus, after a successful conjugation and subsequent triggered release of MTX, we understand that SPION@PGlCLCys has a strong potential to be treated as a model nanoplatform for the development of treatments and diagnosis techniques (or theranostic applications) that can be less aggressive to patients.

## 1. Introduction

Traditional cancer treatments typically involve the use of non-selective agents, that besides action on destroying the tumor cells, also damage healthy tissues and organs, causing side effects that many times can be life threatening [1]. The use of nanotechnology for cancer treatment allows the engineering of nanostructures for specific targeting, together with the controlled release, being able to reach places of very difficult access inside the human body [2]. Thus, it has been the focus of many research groups around the globe. In this context, superparamagnetic iron oxide nanoparticles (SPIONs) have their use approved for the diagnosis/treatment of malignant tumors and can be metabolized by the organism [3].

To prevent embolism caused by SPIONs, these need to be coated with biocompatible and non-cytotoxic materials [4]. Synthetic polymers, such as non-biodegradable poly (methyl methacrylate), have been used for this purpose. However, polyesters derived from lactones and macrolactones appear as promising alternatives due to their biocompatibility, and biodegradability. Poly (ε-caprolactone) (PCL) is a great example of a biodegradable and biocompatible polyester, which is already applied in commercial biomedical devices [5]. On the other hand, unsaturated macrolactones provide (co)polymers that can be functionalized by thiol-ene “click reactions” with different (bio)molecules, such as amino acids and their derivatives, drugs, proteins, antibodies, etc. [6]. This kind of modification is a topic of great interest in biomedical applications, being useful as tools in targeting breast cancer in both diagnosis [7] and treatment [8,9]. Globalide is an unsaturated macrolactone that is gaining attention as a monomer to obtain unsaturated polyesters of great interest in the medical field [10]. Recent advances in polymerization and modification methods involving unsaturated polyesters have aroused increasing interest, as they allow obtaining polymers with modified characteristics and properties, such as lower crystallinity, and higher hydrophilicity, in addition to specific interactions with specific cells, for example [11,12,13,14].

Cysteine (Cys) is an amino acid that was reported to interact on the surface of the SPIONs through carboxylic acid, thiol, and amine groups [15]. Therefore, the modification of unsaturated polyesters with Cys is a promising strategy, since the cysteine-modified polyester can be applied in the coating of SPIONs, given the good compatibility between Cys and the SPIONs. Furthermore, the presence of Cys on the surface of the SPIONs provide amine groups available for further modification, which would give a specific function to the nanoparticle. Additionally, cysteine-modified polyesters tend to present low crystallinity and high hydrophilicity, which would increase its degradability, as previously reported in polymer modification studies using N-acetylcysteine [16].

There are many examples of (bio) molecules that could be conjugated to the amine groups present in Cys, giving a specific function to the SPIONs. Folic acid (FA) is a naturally occurring molecule and an essential nutrient for humans [17]. FA can be used as a targeting agent to cancer cells, since several tumor cells overexpress FA receptors. Thus, the use of FA for surface functionalization promotes cellular internalization of SPIONs [18]. In this way, the mechanism of endocytosis mediated by the folate receptor and the subcellular traffic of nanoparticles [19] could be achieved. Methotrexate (MTX) is another example. MTX is an anti-cancer drug that also has specific interaction for folate receptors in tumor cells, and acts blocking the body’s use of FA, which is responsible for the rapid division of cells, thus controlling the growth of the tumor [20].

**Scheme 1:**
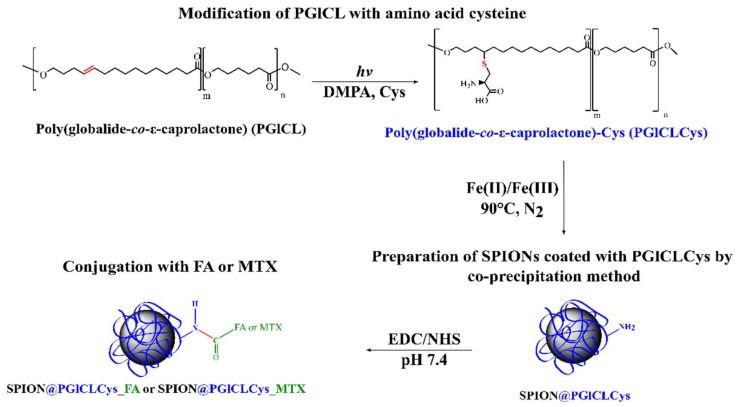
Synthesis of PGlCLCys, preparation of SPIONs, and conjugation with either folic acid (FA) or methotrexate (MTX).

In this context, the present work (Scheme 1) is focused on the preparation of SPIONs coated by an unsaturated copolymer poly(globalide-*co*-caprolactone) (PGlCL) modified with cysteine (SPION@PGlCLCys). Cysteine, besides providing the proper protection of the SPION core, offers important amine groups as anchoring points for further conjugation with FA or with MTX. FA and MTX were used (separately) for the production of conjugates (SPION@PGlCLCys_FA and SPION@PGlCLCys_MTX) focused on applications for cancer treatment/diagnosis. Both molecules were conjugated by amidation reaction between their carboxylic acid and the amine group of cysteine. Release assays for MTX were performed in the presence of a protease, since proteases, that can cleave amide bonds, are present in the lysosome of the tumor cells [21,22]. Cell viability assays (MTT assay) were also carried out in order to evaluate the performance of the conjugate SPION@PGlCLCys_MTX in acting against tumor cells (MDA-MB 231). To the best of our knowledge, this is the first report on the preparation of a nanoplatform made of SPION@PGlCLCys, which has a multifunctional purpose and has a strong potential to be applied in targeted drug delivery, hyperthermia, diagnosis of cancer, or even in theranostics.

## 2. Materials and Methods

### 2.1. Materials

Acetone P.A 99.8%, chloroform P.A. 99.8%, dichloromethane P.A. 99.8% (DCM), dimethylformamide P.A 99.5%, dimethyl sulfoxide P.A 99.7%, ethanol P.A. 99.8% (EtOH), tetrahydrofuran P.A. 99.8% (THF), and toluene P.A. 99.0% were purchased from Merck (Rio de Janeiro, Brazil). The photoinitiator 2,2-dimethoxy-2-phenylacetophenone (DMPA) CAS:24650-42-8 was kindly donated by IGM resins (Valinhos, Brazil). Iron (III) chloride hexahydrate (FeCl_3_∙6H_2_O), iron (II) chloride tetrahydrate (FeCl∙4H_2_O), and ammonium hydroxide (NH_4_OH) were purchased from Vetec (Duque de caxias, Brazil). Folic acid (FA) 98%, 1-ethyl-3-(3-dimethyl aminopropyl) carbodiimide hydrochloride (EDC), N-Hydroxysuccinimide (NHS), methotrexate hydrate (MTX), fluorescein isothiocyanate (FITC), and enzyme Bromelain were purchased from Sigma-Aldrich (Cotia, Brazil). Cysteine hydrochloride 99.8% (Cys) was purchased from Gemini (Anápolis, Brazil). Novozym 435 (commercial lipase B from Candida Antarctica immobilized on cross-linked polyacrylate beads) was kindly donated by Novozymes, Brazil, A/S. Globalide (Gl) was a kind gift from Symrise (Cotia, Brazil), while ε-caprolactone (CL) was purchased from Sigma-Aldrich (Cotia, Brazil). Globalide and ε-caprolactone were dried under vacuum for 24 h and kept in a desiccator over silica and 4 Å molecular sieves. Water was purified by a Milli-Q water purification system.

### 2.2. Experimental Procedure

#### 2.2.1. Enzymatic Synthesis of Poly(Globalide-*co*-ε-caprolactone)

The synthesis of PGlCL was carried out according to the method used by Guindani et al. [23], with modifications. The polymerization was performed in a 50/50 mass ratio (Gl/CL). Toluene was used as solvent (toluene:monomers = 1:2, *w*/*w*), and the system was maintained at a temperature of 65 °C for 2 h. The enzyme content was fixed at 5 wt.% relative to the total mass of monomer. After polymerization, the immobilized enzyme (N-435) was filtered off and the copolymer was precipitated in a mixture of cold EtOH: acetone (70:30 v:v), being dried under vacuum up to constant weight.

#### 2.2.2. Modification of PGlCL with Cysteine via Thiol-Ene Reaction

The post-polymerization modification of PGlCL was carried out by photopolymerization using thiol-ene reactions using a DMPA photoinitiator, directly on PGlCL unsaturations [24]. Cysteine was chosen as a functionalizing molecule because it contains a thiol group and because its presence as a pendent group on PGlCL chains confers desirable hydrophilic characteristic and, furthermore, enables the covalent conjugation of high interest biological interest molecules to the nanoparticle surface. For the modification procedure, the copolymer PGlCL (0.300 g) and Cys (0.224 g) were placed in a flask with the photoinitiator DMPA (0.016 g), using a mixture of chloroform (4 mL) and DMF (2 mL) as a solvent, under nitrogen atmosphere. The reaction was carried out in a UV chamber for 4 h, under continuous magnetic stirring. The amount of Cys used was established to be twice the minimum required to react with all double bonds.

#### 2.2.3. Synthesis of Superparamagnetic Iron Oxide Nanoparticles (SPIONs) and Coating with PGlCLCys

SPIONs were synthesized by the Fe_3_O_4_ co-precipitation method [25] and PGlCLCys was used as the coating agent. First, solutions of FeCl_2_∙4H_2_O (2.0 mmol) and FeCl_3_∙6.H_2_O (4.0 mmol) salts were prepared in the proportion of 1:2 (mol/mol) dissolved in 1 mL of 1M HCl solution under an inert atmosphere, to avoid oxidation of Fe (II). Then, 100 mL of deionized water was heated up to 80 °C, and the solution containing Fe^3+^ and Fe^2+^ was added under nitrogen flow. When the solution reached 90 °C, 40 mL of NH_4_OH (25% v:v) was added, reaching pH~10. Next, the modified copolymer (PGlCLCys) was added (Fe^3+^ PGlCLCys = 8:1 mol/mol), and the system was kept under constant stirring at 90 °C during 1 h. The appearance of a dark brown/black color in the solution is indicative of the formation of coated iron oxide, forming SPION@PGlCLCys. After that time, SPIONs were separated with a magnet and washed with distilled water. Afterwards, the sample was frozen and lyophilized for the subsequent analyses.

#### 2.2.4. Conjugation of SPION@PGlCLCys with Folic Acid

Folic acid was conjugated to SPION@PGlCLCys by the carbodiimide approach [26]. First, FA was activated with NHS for further conjugation with the SPION@PGlCLCys. FA (2 mmol), NHS (2 mmol), and EDC·HCl (2.2 mmol) were dissolved in 100 mL DMSO. The mixture was purged with nitrogen, and the reaction was carried out overnight, under constant stirring, at room temperature, and protected from light. DMSO and unreacted FA were removed by dialysis (Spectra-Por 100-500 Da, Biontech CE Tubing) in a buffer solution (PBS, pH 8.0) for four days, replacing the buffer solution daily. After dialysis, the FA-NHS solution was lyophilized. For the conjugation of FA-NHS to the SPION@PGlCLCys surface, the amount of cysteine in the copolymer was calculated based on the consumption of the double bonds of the copolymer, determined by ^1^H NMR analysis. The proportion between carboxylic acid groups (from FA-NHS) and amine groups (from PGlCLCys) was varied: it was tested a stoichiometric ratio of NH_2_:COOH = 1:1; and an excess of carboxylic groups (FA-NHS), where with a ratio of NH_2_:COOH = 1:2. In sequence, 5 mg of SPION@PGlCLCys was dispersed in phosphate buffer (pH~8.0) with the help of a sonicator. After the complete dispersion of the SPION@PGlCLCys, the amounts of FA-NHS 0.160 mg (NH_2_:COOH = 1:1) and 0.260 mg (NH_2_:COOH = 1:2) were added to falcon tubes containing SPION@PGlCLCys. The system was purged with nitrogen and left in a Klein-type shaker for 24 h. Afterwards, magnetic separation was performed and samples were submitted to 3 washing cycles, collecting the supernatant for FA quantification, and calculation of the conjugation efficiency. The amount of FA conjugated to the SPION@PGlCLCys was calculated based on a FA calibration curve (λ = 283 nm) using UV–vis equipment (Perkin Elmer, Shelton, CT, USA).

#### 2.2.5. Conjugation of SPION@PGlCLCys with Methotrexate

For the conjugation of the drug MTX to the surface of SPION@PGlCLCys, the same approach described in Section 2.2.4 for the conjugation of SPIONs with FA was applied. The amount of MTX conjugated to SPION@PGlCLCys was calculated based on an MTX calibration curve (λ = 303 nm) using UV–vis equipment.

### 2.3. Physicochemical Characterizations

Proton nuclear magnetic resonance (^1^H NMR) analyses were performed on Bruker AC-200F NMR equipment, operating at 200 MHz. Chemical shifts are reported in ppm relative to tetramethylsilane (TMS) 0.01% (vol%) (δ = 0.00). All samples were dissolved in CDCl_3_ (δ = 7.27 for 1H NMR). Poly(globalide-*co*-ε-caprolactone) ^1^H NMR (CDCl_3_ 200 MHz): δ(ppm) 5.49–5.32 (m, CH=CH); 4.10–4.04 (m, CH_2_O(C=O)). Poly(globalide-*co*-ε-caprolactone) -Cys RMN ^1^H (CDCl_3_ 200 MHz): δ(ppm) 5.49–5.32 (m, CH=CH); 4.10–4.04 (m, CH_2_O(C=O)); 2.90–2.70 (m, S–CH_2_). Molecular weight distributions were determined by Gel Permeation Chromatography (GPC). Therefore, 0.02 g of the copolymer was dissolved in 4 ml of tetrahydrofuran (THF) and the solution obtained was filtered (pore: 0.45 µm, diameter: 33 mm) before the analysis. The analysis was performed using a high-performance liquid chromatography equipment (HPLC, model LC 20-A, Shimadzu, Kyoto, Japan) and Shim Pack columns of the GPC800 series (GPC 801, GPC 804, and GPC 807), also from Shimadzu. As eluent, THF was used at a volumetric flow rate of 1 mL min^−1^ at 40 °C. Calibration was achieved using polystyrene standards. Polystyrene standards with molecular weights ranging from 580 to 9.225 × 10^6^ g·mol^−1^ were used to construct the standard curve. Fourier transform infrared spectroscopy (FTIR) was used to identify the chemical structure of the cysteine-modified copolymer and SPION@PGlCLCys. The analysis was performed on a Prestige 21 spectrophotometer (Shimadzu IR, Kyoto, Japan) using the KBr tableting technique for obtaining a transparent tablet. The spectra were recorded in a wavenumber range of 400 to 4000 cm^−1^. Differential scanning calorimetry (DSC) was used to measure melting temperatures and melting enthalpy, used to determine the degree of crystallinity of the polymeric materials using a Jade DSC (Perkin-Elmer, Shelton, CT, USA). For the analysis, approximately 5 mg of the dried polymer was analyzed under nitrogen atmosphere (20 mL·min^−1^), with temperatures ranging from 0 to 150 °C and a heating rate of 10 °C min^−1^. The thermal history was removed before the analyses (for pure polymer samples) at a heating rate of 20 °C min^−1^ and a cooling rate of 10 °C min^−1^. The second heating runs were used to obtain the thermal properties of the polymer. For the contact angle assay, PGlCL and PGlCL-Cys films were produced, by depositing copolymer solutions (100 mg:mL—Chloroform:DMF 9:1) on microscope slides. The contact angle between the polymeric films and the water drops was measured in a goniometer Ramé-Hart 250 (Ramé-Hart Instrument Co., Succasunna, NJ, USA). For this assay, samples were analyzed in triplicate at room temperature, using 10 μL drop volume. Transmission electron microscopy and selected area electron diffraction (TEM/SAED) images were obtained on a JEM-1011 TEM (JEOL, Peabody, MA, USA) microscope at an acceleration voltage of 100 kV. Powder X-ray diffraction (XRD) analyses were performed on a Rigaku MiniFlex 600 (Rikagu, Tokyo, Japan) diffractometer with graphite monochromatized CuKα (λ = 1.5418 A°), with maximum voltage and current at 40 kV and 40 mA, respectively, with a 2θ°/min scan rate in the range of 20 to 80° with 0.05° steps. Magnetic properties of SPION@ PGlCLCys were measured in a vibrating sample magnetometer (VSM) EV9 Model (MicroSense, Lowell, MA, USA). For the VSM analysis, the samples were dried, pressed, and held in a quartz cylinder holder. Thermogravimetric analysis (TGA) was performed under a nitrogen atmosphere at a heating rate of 10 °C min^−1^ from room temperature to 900 °C, in a STA 449-F3 Jupiter (2012) (Netzsch, Hanau, HE, Germany) equipment. The particle size distribution was measured by dynamic light scattering (DLS) and zeta potential using a Zetasizer 3000 HSA (Malvern Instruments, Malvern, Worcs, United Kingdom).

### 2.4. Cell Culture

All biological assays used normal L929 and breast carcinoma-derived MDA-MB 231 cells lines. L929 and MDA-MB 231 were cultivated in RPMI 1640 medium (GIBCO, Baltimore, MD, USA) supplemented with fetal bovine serum (10%), penicillin (100 U/mL) (GIBCO, Baltimore, MD, USA), streptomycin (100 μg/mL) (GIBCO, Baltimore, MD, USA), and glutamine 2 mM. All cells were maintained under controlled conditions (37 °C in a 5% CO_2_ atmosphere with 95% air humidity) for 24 and 72 h. Results are expressed as an average of three independent experiments.

#### 2.4.1. In Vitro Cell Viability of the SPION@PGlCLCys, SPION@PGlCLCys_FA, and SPION@PGlCLCys_MTX

The cell viabilities of L929 and MDA-MB 231 exposed to SPION@PGlCLCys and SPION@PGlCLCys_FA were measured using the MTT [27] assay. For this, 7.5 × 10^3^ cells/well (24 h assay) and 5.0 × 10^3^ cells/well (72 h assay) were seeded in 96-well plates. Following the same procedure, the cell viability of MDA-MB 231 was also evaluated when exposed to SPION@PGlCLCys_MTX and free MTX, for 72 h. At confluence, cells were exposed to different free MTX (8.52 × 10^−6^, 8.52 × 10^−5^, 8.52 × 10^−4^, 8.52 × 10^−3^, 8.52 × 10^−2^, 8.52 × 10^−1^, and 8.52 µg·mL^−1^) and nanoparticle (0.0001, 0.001, 0.01, 0.1, 1, 10, and 100 µg·mL^−1^) concentrations and incubated at 37 °C and pH 7.4 for 24 h and 72 h for each cell. After incubation, cells were washed twice with PBS and incubated for 2 h with MTT (0.5 mg·mL^−1^). Subsequently, the formed formazan crystals were dissolved by the addition of DMSO (100 µL/well), and colored solutions were read at 570 nm. For all tested conditions, the respective blanks were analyzed. The experiments were performed independently for each cell line and in triplicate, and the results are presented as cell viability.

#### 2.4.2. Enzymatic Release of Folic Acid and Methotrexate

The release of the FA conjugated to the SPION@PGlCLCys was studied under the lysosomal condition (pH = 5.3), taking 400 μg of SPION@PGlCLCys_FA and 400 μg of Bromelain protease in 2 mL of PBS (0.1 M solution, pH = 5.3) at 37 °C. The FA release was evaluated for 12 h, 24 h, 48 h, and 72 h [28]. Magnetic separation was performed and the samples were submitted to 3 washing cycles, collecting the supernatant for FA quantification [28]. The amount of released FA was calculated based on a FA standard curve built using UV–vis (283 nm) equipment. The same approach was applied for the release of MTX and the amount of MTX released was calculated based on an MTX standard curve constructed using a UV–vis instrument (303 nm).

### 2.5. Computational Section

The theoretical values of the logarithm of partition coefficient (log P) obtained in the present work were based on the Density Functional Theory (DFT) [29,30]. The simulated molecules were generated and analyzed by the software Avogadro [31] version (1.2.0).

First, the molecular geometry of PGlCL, PGlCLCys, FA, and PGlCLCys_FA were optimized to the ground state geometry of these molecules in different media such as gas phase, n-octanol and water. The optimized structures were confirmed as real minima by vibration analysis (no imaginary frequency was detected) [31]. Through the thermodynamic properties obtained by the DFT calculations, it is possible to obtain the Gibbs free energy in different media and the partition coefficient.

The partition coefficient (P) can be defined as the ratio between the concentration of a solute in two phases of a mixture that contains two immiscible solvents at equilibrium [32]. The logarithm of partition coefficients for n-octanol/water mixtures (log P^O/W^) can be obtained according to [33]:

(1) $$\log P^{O/W}=\frac{\Delta G_{solv}^{W}-\Delta G_{solv}^{O}}{2.303RT}$$

where ΔG*solv* = G^X^ − G is the Gibbs free energy of solvation, G and G^X^ are respectively the Gibbs free energy in gas phase and in the solvent. The superscripts (X = W and X = O) represent respectively the water and n-octanol solvents, while R is the ideal gas constant (8.314 J·K^−1^ mol^−1^).

The results were obtained at the reaction conditions (25 °C and 1.0 atm), using the Becke-three-parameter Lee–Yang–Parr (B3LYP) model as hybrid functional, along with a split-valence double-zeta polarized basis set, based on Gaussian type orbitals (6-31G**) [34]. The Gibbs free energy of solvation in water and n-octanol were computed using the solvation model based on electronic density (SMD) [35]. All DFT calculations were performed using the ORCA 5.0.2 package [36].

## 3. Results and Discussion

Scheme 1 shows the steps involved in the development of the nanoplatform. In a first stage, the copolymer PGlCL was synthesized via enzymatic ring-opening polymerization (e-ROP). Subsequently, the modification of the copolymer with the amino acid cysteine was carried out through photoinitiated thiol-ene reactions. The modified copolymer PGlCLCys was then used for the coating of the SPIONS, and the amine groups from cysteine in the SPION@PGlCLCys allow the conjugation of either FA or MTX via carbodiimide chemistry.

### 3.1. Synthesis, Modification, and Characterization PGlCLCys

The synthesis of PGlCL (50:50) was carried out via e-ROP using toluene as solvent at a fixed mass ratio of 2:1 (monomer:solvent). The number (Mn) and weight (Mw) average molecular weights of PGlCL determined by gel permeation chromatography (GPC) were 24.500 g·mol^−1^ and 57.000 g·mol^−1^, respectively. In sequence, the copolyester was modified with cysteine via thiol-ene reaction by photoinitiation with DMPA (PGlCLCys).

**Figure 1:**
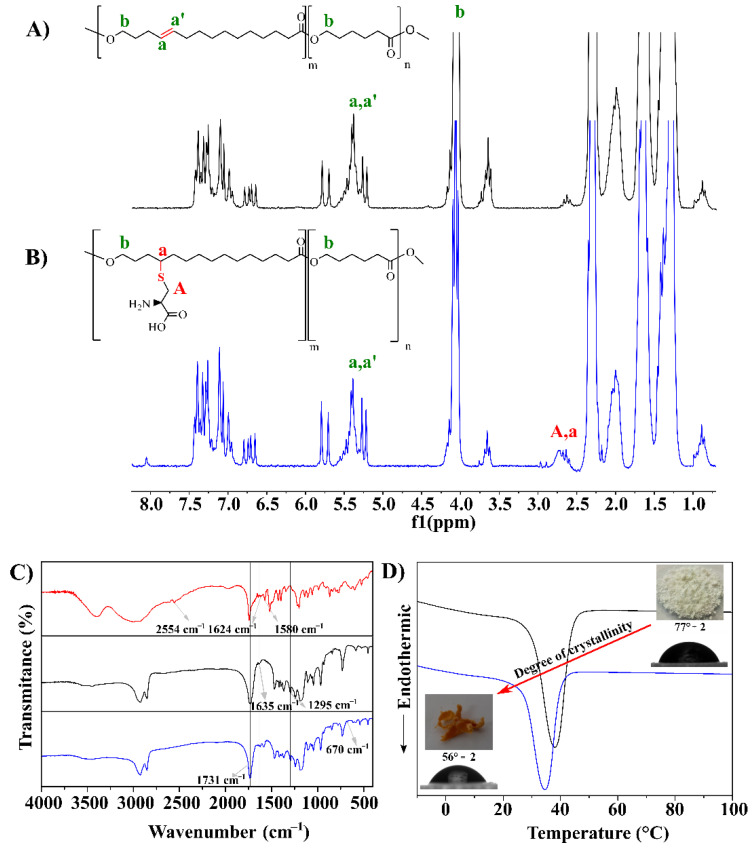
(**A**) ^1^H NMR spectra of PGlCL with peak assignments (a, a’, b). (**B**) ^1^H NMR spectra of PGlCLCys (50/50 Gl/CL weight ratio) with peak assignments (a, a’, b, a, A). (**C**) FTIR spectra of cysteine (red) and respective copolymers PGlCL (black) and PGlCLCys (blue). (**D**) Second run DSC heating curves of PGlCL (black) and PGlCLCys (blue) with different respective contact angle.

The modification of the polymer with cysteine was verified by FT-IR (Figure 1A). For PGlCL a band was observed in 1635 cm^−1^ referring to the elongation of the alkene group (–C=C–) present in the PGlCL. The same band could not be observed in the PGlCLCys spectrum. Additionally, the appearance of bands at 674 cm^−1^ (–C–S–C–) and at 1580 cm^−1^ (–C–NH_2_) confirms the incorporation of cysteine to the copolymer. The nonappearance of bands at 2550 cm^−1^ (–C–SH) indicates the absence of free cysteine in the modified polymer.

The degree of modification of PGlCL with cysteine was determined by ^1^H NMR analysis (Figure 1B) based on the peak area of the double bond (–C=C–, at 5.40 ppm) present in globalide repeat units in the copolymer (results in Table S1, see Supplementary Materials (SM)). For integration, the peak of ester methylene at 4.06 ppm was used as a reference, since the amount of ester methylene remains constant. An 18% consumption of double bonds was observed after the reaction with cysteine, indicating the coupling of cysteine to 18% of the globalide repeating units of PGlCL. The modification was also evidenced by the emergence of a peak at 2.90–2.70 ppm, that can be assigned to the –S–CH_2_– group. It is important to emphasize that due to the hydrophobic character of PGlCL and the hydrophilic character of cysteine hydrochloride, solubilizing both reactants simultaneously is a great challenge, and it hinders the modification reaction. However, in spite of the relatively low degree of modification, the success of the modification reaction is evidenced by the changes in physicochemical properties of the copolymer, reported below.

The degree of crystallinity was determined by the relationship between the ΔH*_m_* measured by DSC of each sample and the theoretical value of a 100% crystalline PCL sample (more details in Table S2 in SM). The melting temperature (T*_m_*) for PGlCL was 38 °C and after modification with cysteine, T*_m_* was reduced to 32 °C. At the same time, the crystallinity decreased from 44% to 30% after the reaction with the amino acid (Figure 1C). The modification of the polyester with cysteine also affected its hydrophobic character, reducing the value of the contact angle with water from 77° to 56° (Figure 1C). The decrease in the melting point and in the degree of crystallinity are related to the presence of cysteine as a pendant group in the polymer chains. Cysteine acts as a “spacer” molecule, which disturbs the crystalline arrangement, consequently decreasing the degree of crystallinity. The decrease in the melting temperature, in turn, is due to the increase in free volume caused by the presence of cysteine, which reduces the energetic level necessary to overcome the secondary intermolecular forces between the chains of the crystalline phase [37]. The increase in the hydrophilicity of the polymer, on the other hand, is endorsed by the prediction of the partition coefficient (P, also represented in the logarithm form, log P) through DFT calculations (for details, see Table S3 on SM). The partition coefficient is defined as the ratio between the concentration of a solute in two phases of a mixture of two immiscible solvents at equilibrium (in this case, n-octanol and water, log P^O/W^) [32], being a form to evaluate the hydrophobicity or hydrophilicity of a solute. The polymer PGlCL presented log P^O/W^ = 5.53, while after the modification with cysteine, this value decreased to log P^O/W^ = 3.54, indicating an increase in hydrophilicity, which is mostly affected by the polar character of the carboxyl group [37,38].

### 3.2. Preparation of SPIONs Coated with PGlCLcys by Co-Precipitation Method

**Figure 2:**
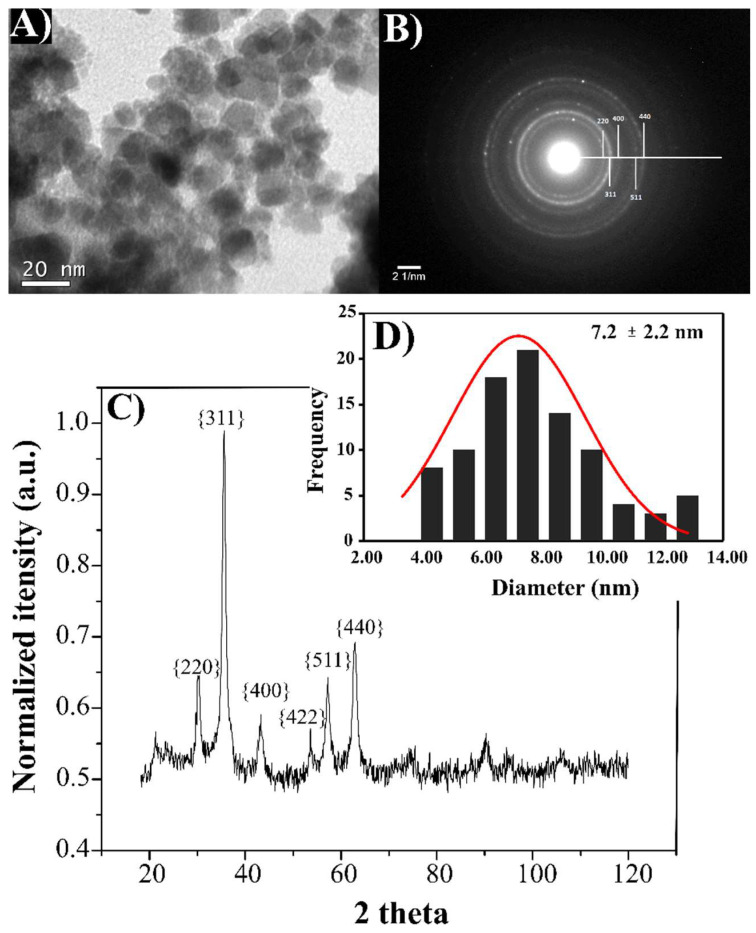
SPION@PGlCLCys (**A**) Low magnification TEM image; (**B**) SAED; (**C**) XRPD diffractogram with diffraction peaks indexed to the spinel iron oxide phase. (**D**) Particle size distribution of the magnetic nanoparticles obtained from the TEM images.

The preparation of SPIONs coated with PGlCLcys (SPION@PGlCLCys) was performed according to the co-precipitation approach with modifications [25,39]. The SPION@PGlCLCys exhibited an average particle diameter of 7.2 ± 2.2 nm (Figure 2A,D), determined by transmission electron microscopy (TEM-100 kV), and a monomodal distribution, as well as high crystallinity (Figure 2B) determined by selected area electron diffraction (SAED) technique. The average crystalline domains and core size were around 7.6 nm as estimated from the results of X-ray powder diffraction (XRPD) analysis (Figure 2C). The lattice parameter obtained after Rietvield refinement (a = 8.36154 Å) applied to the XRPD data (0.252 nm to (311)) is consistent with the value of 8.46 Å based on the (220) interplanar spacing of 0.299 nm obtained using SAED (Figures S3 and S4, see SM). Thus, the results agree with those of other studies [40] related to the spinel structure of Fe_3_O_4_ and standard measurements (a = 8.3940 Å) (for more details, see Table S4, in SM).

The superparamagnetic behavior of SPION@PGlCLCys observed by VSM analysis corroborates the core size below 15 nm [41] and the successful coating of the SPIONs, presenting a saturation magnetization of 60 emu·g^−1^, which is consistent with saturation magnetization values obtained for magnetite in literature [42,43] (for details, see Figure S7, in SM). The coating of the SPIONs by PGlCLCys was also evidenced by the FTIR spectra (Figure S5, in SM), indicated by vibration frequencies at 1736 cm^−1^ (elongation –C=O), associated with a carboxyl group and elongation of the amine group at 1643 cm^−1^ (–NH_2_).

TGA results (Figure S6) gives important information on the SPION@PGlCLCys composition. The thermogram shows a mass loss of 1.7%, related to water evaporation, followed by a mass loss of 13.3%, assigned to PGlCLCys decomposition, and finally the 85% that remained corresponds to the iron oxide presence. The proportion of Fe3O4:PGlCL used in the preparation of the SPION@PGlCLCys was 8:1 (*w*/*w*), which means theoretically there are 11.1% PGlCLCys and 88.9% Fe_3_O_4_ in SPION@PGlCLCys. Therefore, one can say that the experimental results is in accordance with the theoretical composition values.

SPION@PGlCLCys were resuspended in buffer solution (pH = 8.0, close to physiological conditions) and Dynamic Light Scattering (DLS) measurements were performed (see Figure S8, in SM). The intensity average particle diameter measured was 145 nm with a PDI of 0.2. This result shows that after being dispersed, SPION@PGlCLCys formed some aggregates, but the unimodal size distribution and PDI value of 0.2 indicate that such aggregates are stable. A possible cause for this aggregation is the small amount of PGlCLCys used to coat the SPIONs, which provides a very thin layer of polymer coating the magnetite core. On one hand, this has a very positive impact on the saturation magnetization of SPION@PGlCLCys (60 emu·g^−1^). On the other hand, such a thin layer does not avoid a strong interaction between the nuclei and provides some aggregation of SPIONs after the lyophilization process. However, it is also important to emphasize that hydrodynamic diameter of 145 nm with a PDI of 0.2, which is an acceptable size for drug delivery purposes. The SPION@PGlCLCys also showed a zeta potential value of −35.4 mV (pH = 8.0, close to physiological conditions), suggesting moderate colloidal stability [44]. Such negative value is much likely related to the presence of cysteine in the polymer, suggesting that in addition to developing the role of protecting the SPIONs core, PGlCLCys also helps in some extent to maintain some colloidal stability in the system. The isoelectric point of the amino acid cysteine is widely reported in literature as being 5.07 [45]. That is, in pH values above 5.07, cysteine structure is deprotonated, assuming an overall negative charge.

### 3.3. Conjugation of FA or MTX on the SPION@PGLCLCys, Enzymatic Release, and Cell Viability

Since SPION@PGlCLCys is stable in aqueous medium, its surface was covalently conjugated with FA by carbodiimide approach, aiming to target the folate receptors in breast cancer. The amount of FA covalently bonded to the SPION@PGlCLCys was determined by UV–vis spectrometry, by using a FA calibration curve, dissolved in pH = 8.0 buffer at 286 nm (further details see Figure S10A, in SM). Considering NH_2_:COOH ratios of 1:1 (stoichiometric proportion) and 1:2 (excess of FA), the conjugation efficiency of FA on the SPIONs surface was 62% and 183.5%, respectively, relative to the available NH_2_ sites for conjugation. A conjugation efficiency higher than 100% is explained by the adsorption of the FA in excess on the surface of the SPION@PGlCLCys. For the release assays and cell viability assays, we chose to proceed with the samples synthesized using a NH_2_:COOH ratio of 1:1, containing approximately 8.27 μg FA/mg iron oxide.

DFT calculations were also performed in order to provide theoretical information on the hydrophilicity of PGlCLCys_FA, which presented an estimated log P^O/W^ value of 0.81, a much lower value than PGlCL (log P^O/W^ = 5.53) and PGlCLCys (log P^O/W^ = 3.54), indicating that the presence of FA makes SPION@PGlCLCys_FA quite hydrophilic. Such hydrophilic characteristic must be highlighted, since the hydrophilization of nanoparticles is commonly reported as a strategy to increase their circulation time in the blood stream [46].

The cytotoxicity of SPION@PGlCLCys and SPION@PGlCLCys_FA was evaluated by the MTT assay using L929 and MDA-MB 231 cell lines (for details, see Figures S11 and S12 in SM). Cell viability reached 100% for all tested samples, in the concentration range from 1.10^−4^ ppm to 100 ppm, for 24 h and 72 h, and for both tested cell lines. In sequence, the release profile of conjugated FA on the surface of SPION@PGlCLCys_FA was evaluated. This way, bromelain was used as a protease to simulate a possible release triggered via lysosomal protease at 37 °C and pH~5.3, similar to cellular pH, to verify the cleavage of the amide bond formed in the conjugation of FA to the surface of SPION@PGlCLCys (more details, see Figure S13, in SM). Results showed that protease cleaves approximately ~28% of the amide bonds within 24 h, and ~35% in 72 h. This is the main route of drug release, since for the assay at the same pH but without enzyme, there is no release.

Finally, the conjugation of the drug MTX on SPION@PGlCLCys was performed, and the conjugation efficiency was determined by the UV–vis method (calibration curves in Figure S10, SM, dissolved in pH = 8.0 buffer, at 303 nm). The results obtained for MTX conjugation efficiency are 60.3% (1:1 stoichiometric proportion) and 132.4% (1:2 excess of MTX), relative to the available NH_2_ sites for conjugation. A conjugation efficiency higher than 100% is explained by the adsorption of the MTX in excess on the surface of the SPION@PGlCLCys Again, for the release assays and cell viability assays, we chose to proceed with the samples synthesized using a NH_2_:COOH ratio of 1:1, which provided the highest conjugation efficiency, containing approximately 3.20 μg MTX/mg iron oxide.

**Figure 3:**
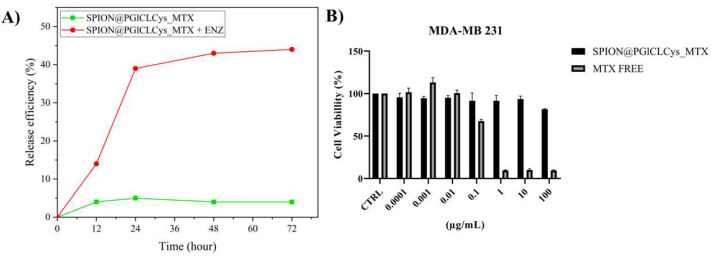
SPION@PGlCLCys_MTX: (**A**) drug delivery assay at lysosomal pH (pH 5.3) with or without protease (ENZ); (**B**) breast carcinoma-derived MDA-MB 231 cells viability after 72 h at different MTX concentrations.

Similar to FA, the enzymatic release assay for SPION@PGlCLCys_MTX was carried out at lysosomal pH. The results (Figure 3A) showed that protease cleaves approximately ~38% of the amide bonds within 24 h, and ~45% in 72 h. This can be considered a very positive result, since other works in literature that investigated MTX encapsulation in polyesters nanoparticles (e.g., polycaprolactone) often report a release of around 40% in 72 h, or even less depending on how hydrophobic the polymer matrix is [47,48]. Therefore, it is important to emphasize that in our conjugate (SPION@PGlCLCys_MTX), MTX is not only acting as a drug itself, but also as a molecule that has specific interactions for folate receptors, promoting specific targeting in tumor cells, forming the basis of our proposal of a multifunctional nanoplatform for cancer treatment.

Regarding the MTT cell viability assay, for 72 h incubation, a strong reduction in breast carcinoma-derived MDA-MB 231 cell viability was observed in free MTX for concentrations above 0.1 ug·mL^−1^. For the conjugates SPION@PGlCLCys_MTX, this decrease was smoother reaching a reduction of approximately 20% at the highest evaluated concentration (Figure 3B).

The superior behavior of free MTX in comparison to SPION@PGlCLCys_MTX in the cell viability assays was expected, since free MTX is immediately available to interact with the cells, while MTX attached to SPION@PGlCLCys releases about 45% of MTX in 72 h.

## 4. Conclusions

In this work, we have successfully prepared SPIONs coated with a polyester modified with the amino acid cysteine (SPION@PGlCLCys). The cysteine molecules present in the SPIONs’ coating can be used as anchoring points for the conjugation of a wide variety of molecules (e.g., peptides, antibodies, drugs, etc.). The main goal here was to prepare SPION@PGlCLCys and test their potential as a multifunctional nanoplatform for future developments to the traditional cancer treatments. In order to test such potential, we conjugated the SPION@PGlCLCys with two different molecules, separately, in order to understand their behavior when conjugated to SPION@PGlCLCys. FA was chosen due to its characteristic of stablishing specific interactions to folate receptors (overexpressed in tumor cells), as well as methotrexate, an anti-cancer drug that also has specific interaction for folate receptors and acts controlling the growth of the tumor. Both FA and MTX presented satisfactory conjugation efficiencies (above 65%). Release assays were performed in the presence of a protease, and MTX presented 45% release after 72 h, which is a very positive result in comparison to other works that studied the encapsulation and release of MTX from polymeric nanoparticles. MTT assay revealed that after 72 h, the conjugates are capable of reducing the tumor cell viability in about 20%, which is also noted as a very positive result, considering the small amount of MTX conjugated to SPION@PGlCLCys. The results obtained in this work can be seen as the first step for the development of a promising nanoplatform that can be easily modified and improved for future applications in less aggressive cancer treatments, allying targeting of tumor cells, controlled drug delivery, hyperthermia, and eventually diagnosis (theranostics).

## Data Availability

The data presented in this study are available on request from the corresponding author.

## Supplementary Materials

The following Supplementary Materials can be downloaded at: https://www.mdpi.com/article/10.3390/pharmaceutics15031000/s1. Figure S1. Representative scheme of the reaction steps to obtain PGlCL and its modification with cysteine followed by coating of SPIONs. A) Enzymatic Ring-Opening Polymerization reaction (e-ROP). B) Modification reaction with the amino acid cysteine (Cys) via thiol-ene reaction to obtain PGlCLCys. C) Synthesis and stabilization of SPIONs with PGlCLCys. D) Conjugation of SPIONs with FA. E) Conjugation of SPIONs with MTX. Figure S2: TEM dark field image showing individual crystalline particles (DF-TEM) of SPION@PGlCLCys. Figure S3: Selected area electron diffraction (SAED) image showing indexed diffraction rings corresponding to magnetite crystallographic planes of SPION@PGlCLCys. Figure S4: SAED image with Indexed diffraction pattern of magnetite and simulated diffraction ring pattern matching the results for the SPION@PGlCLCys sample. Figure S5: FT-IR spectra for SPIONs, and modified copolymer (PGlCLCys) and after coating of magnetic nanoparticles (SPION@PGlCLCys). Figure S6: Thermogravimetric analysis of SPION@PGlCLCys. Figure S7: VSM analysis of SPION@PGlCLCys. Figure S8: DLS analysis of SPION@PGlCLCys. Figure S9: Zeta potential surface analysis of SPION@PGlCLCys (ζ = −35.4 mV) dispersed in buffer solution (pH = 8.0). Figure S10: UV-vis calibration curve for: A) folic acid (FA), and B) methotrexate (MTX) in buffer solution (pH = 8.0). Figure S11: MTT assay of SPION@PGlCLCys and SPION@PGlCLCys_FA showing cells viability as a function of nanoparticles concentration (0.0001 to 100 µg·mL−1) for 24 h. All SPIONs tested at different concentrations did not exert difference (ANOVA) in relations to the control group n = 3. Figure S12: MTT assay of SPION@PGlCLCys and SPION@PGlCLCys_FA showing cells viability as a function of nanoparticles concentration (0.0001 to 100 µg·mL−1) for 72 h. All SPIONs tested at different concentrations did not exert difference (ANOVA) in relations to the control group n = 3. Figure S13: FA assay at lysosomal pH (pH 5.3). Table S1: Thiol-ene reaction conversion calculated based on the consumption of the double bonds present in PGlCL chains, determined by 1H NMR. Table S2: Thermal properties of the polymers, determined by DSC. Table S3: Gibbs free energy calculated at 1 atm and 25 °C using DFT/B3LYP/6-31G** with water and n-octanol solvents in SMD model. Table S4: Interplanar distance.
