# Supplementary material for: Coating of SPIONs with a Cysteine-Decorated Copolyester: A Possible Novel Nanoplatform for Enzymatic Release
Source: Pharmaceutics. 2023 Mar 20;15(3):1000. doi: 10.3390/pharmaceutics15031000 (PMC10058032; doi:10.3390/pharmaceutics15031000)
Supplement: Supplementary file 1 [file pharmaceutics-15-01000-s001.zip › pharmaceutics-2223734-supplementary.pdf]

Coating of SPIONs with a Cysteine-Decorated Copolyester: A Possible Novel Nanoplatform for Enzymatic Release

Jeovandro Maria Beltrame [1,*], Brena Beatriz Pereira Ribeiro [1], Camila Guindani [2], Graziâni Candiotto [3], Karina Bettega Felipe [4], Rodrigo Lucas [5], Alexandre D'Agostini Zottis [6], Eduardo Isoppo [7], Claudia Sayer [1,*] and Pedro Henrique Hermes de Araújo [1]

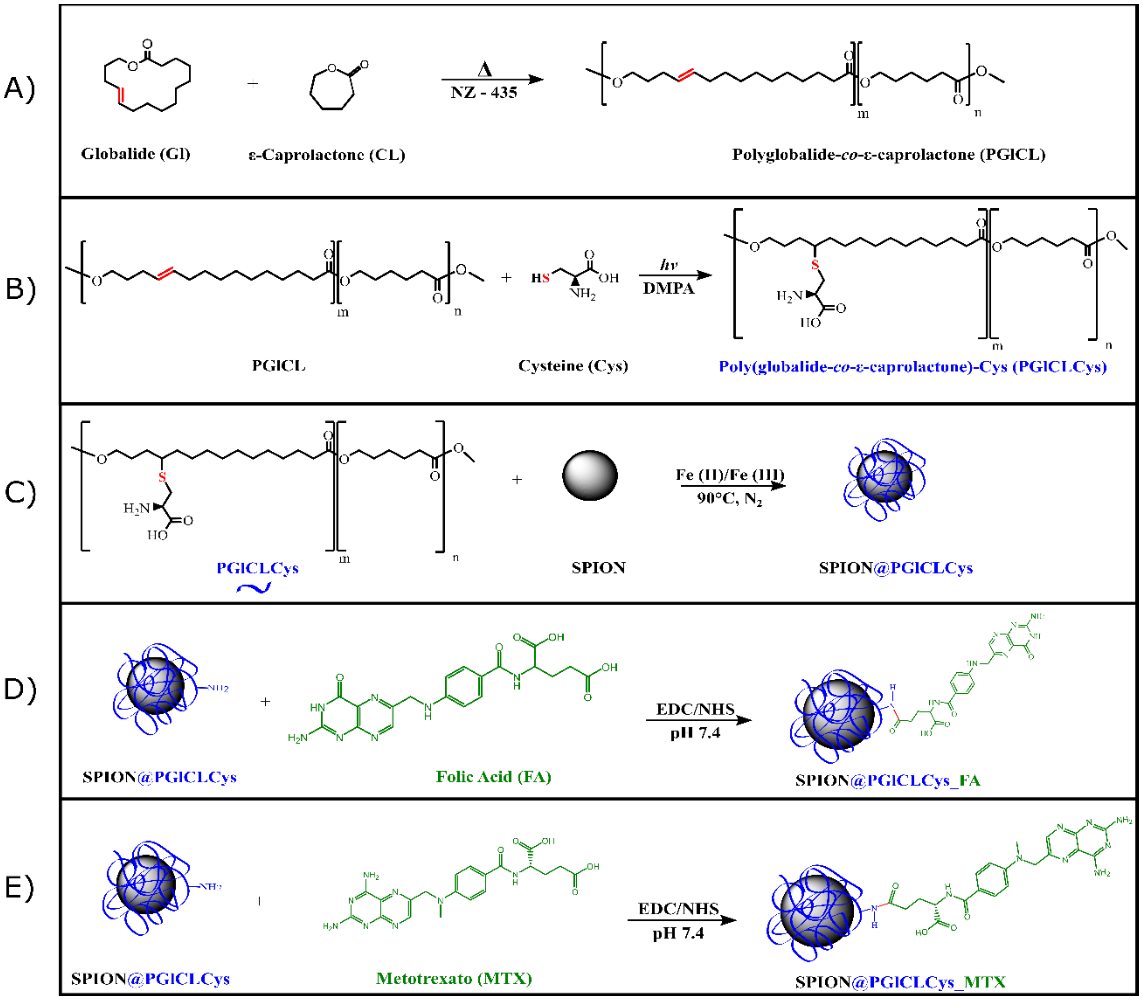

Figure S1. Representative scheme of the reaction steps to obtain PGlCL and its modification with cysteine followed by coating of SPIONs. A) Enzymatic Ring-Opening Polymerization reaction (e-ROP). B) Modification reaction with the amino acid cysteine (Cys) via thiol-ene reaction to obtain PGlCLCys. C) Synthesis and stabilization of SPIONs with PGlCLCys. D) Conjugation of SPIONs with FA. E) Conjugation of SPIONs with MTX.

Table S1. Thiol-ene reaction conversion calculated based on the consumption of the double bonds present in PGlCL chains, determined by ^{1}H NMR.

	Integration by corresponding peak (ppm)			
Sample	Double Bond (5.40 ppm)	Reference (4.06 ppm)	Cysteine (2.90 – 2.70 ppm)	Double bond Conversion (%)
PGlCL	1.00	1.00	-	-
PGlCLCys	0.82	1.00	3.00	18%

Table S2. Thermal properties of the polymers, determined by DSC.

**Sample**	**Tm (°C)**	**ΔH (J/g)**	***Xc*** **(%)**
PGlCL	38	59.22	44
PGlCLCys	32	40.6	30

PS: the degree of crystallinity (*X*c) is calculated based on the heat of fusion of a PCL 100% crystalline sample[1].

Table S3 presents the calculated Gibbs free energy (G) and the Gibbs free energy of solvation (G_{solv}) in gas-phase, water and n-octanol. ΔG_{solv} was calculated as the difference between the Gibbs free energy of solvation and gas-phase Gibbs free energy.

Table S3. Gibbs free energy calculated at 1 atm and 25 °C using DFT/B3LYP/6-31G** with water and n-octanol solvents in SMD model.

**Compound**	**Gas-phase**	**Water**		**n-octanol**	
	G **(Kcal/mol)**	G^{W}_{solv} **(Kcal/mol)**	ΔG^{W}_{solv} **(Kcal/mol)**	G^{O}_{solv} **(Kcal/mol)**	ΔG^{O}_{solv} **(Kcal/mol)**
PGlCL	344.3180	348.5650	4.2473	341.0100	3.3081
PGlCLCys	407.4900	416.9260	9.4357	412.0820	4.5921
PGlCLCys_FA	609.0233	615.9962	6.9728	614.8832	5.8599

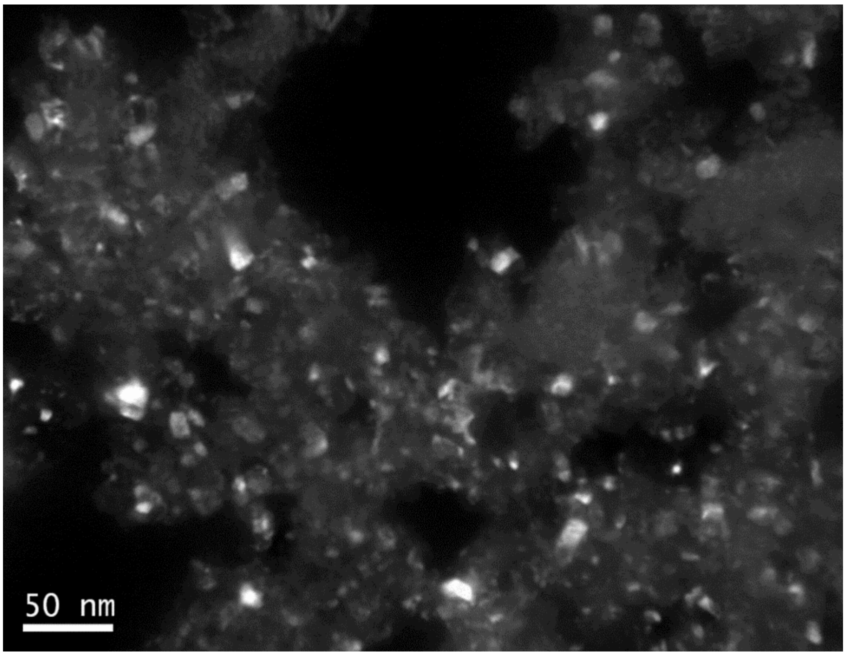

Figure S2. TEM dark field image showing individual crystalline particles (DF-TEM) of SPION@PGlCLCys.

The dark field TEM image (Figure S2) was crucial for the identification of individual nanoparticles, enabling the measurement of the size of nanoparticles. Since the samples tends to agglomerate, the conventional bright field TEM image shows superposed nanoparticles. The dark field technique otherwise makes visible only certain particles with particular geometrical orientation glow facilitating the identification of single-particle boundaries.

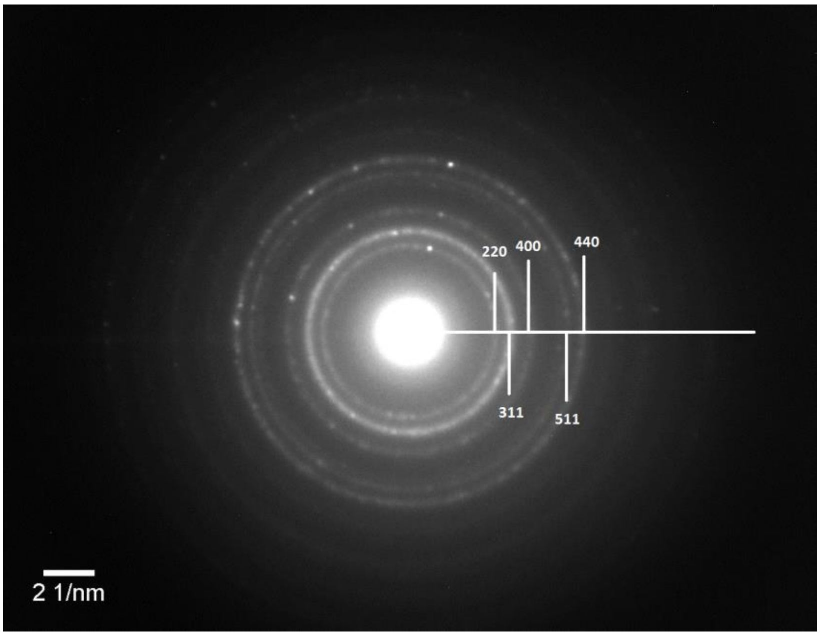

Figure S3. Selected area electron diffraction (SAED) image showing indexed diffraction rings corresponding to magnetite crystallographic planes of SPION@PGlCLCys.

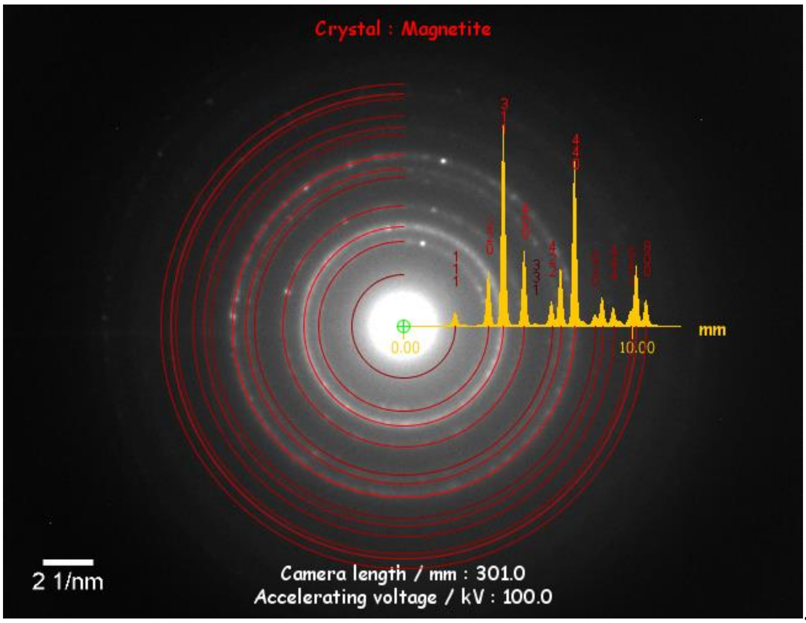

Figure S4. SAED image with Indexed diffraction pattern of magnetite and simulated diffraction ring pattern matching the results for the SPION@PGlCLCys sample.

X-ray diffraction (XRD) analysis was used to characterize the crystallite size, phase, and crystallization of SPIONS. According to the half-maximum full width (FWHM) of (311) reflections, the mean size of the Fe_3O_4 nanocrystalline particles was calculated as 7.662 nm. In addition, the crystal cell dimension of (311) reflections were calculated to be

a = 0.83615 nm using for quantitative analysis the Rietveld approach and the GSAS software.

Table S4. Interplanar distance.

Plane (hkl)	Measured Distance d (nm)	Theoretical Distance[2] d (nm)
220	0.299	0.297
311	0.255	0.253
400	0.212	0.210
511	0.162	0.162
440	0.149	0.148

Table S4 shows the measured interplanar distances obtained by transmission electron microscopy - selected area electron diffraction (TEM–SAED). The values are compared with theoretical values of magnetite[2] and a good agreement is observed.

To calculate the unit cell, the plane distance, calculated with the equation for cubic cells, was used:

$$d_{(hkl)} = \frac{a_0}{\sqrt{h^2+k^2+l^2}} \qquad \text{(S1)}$$

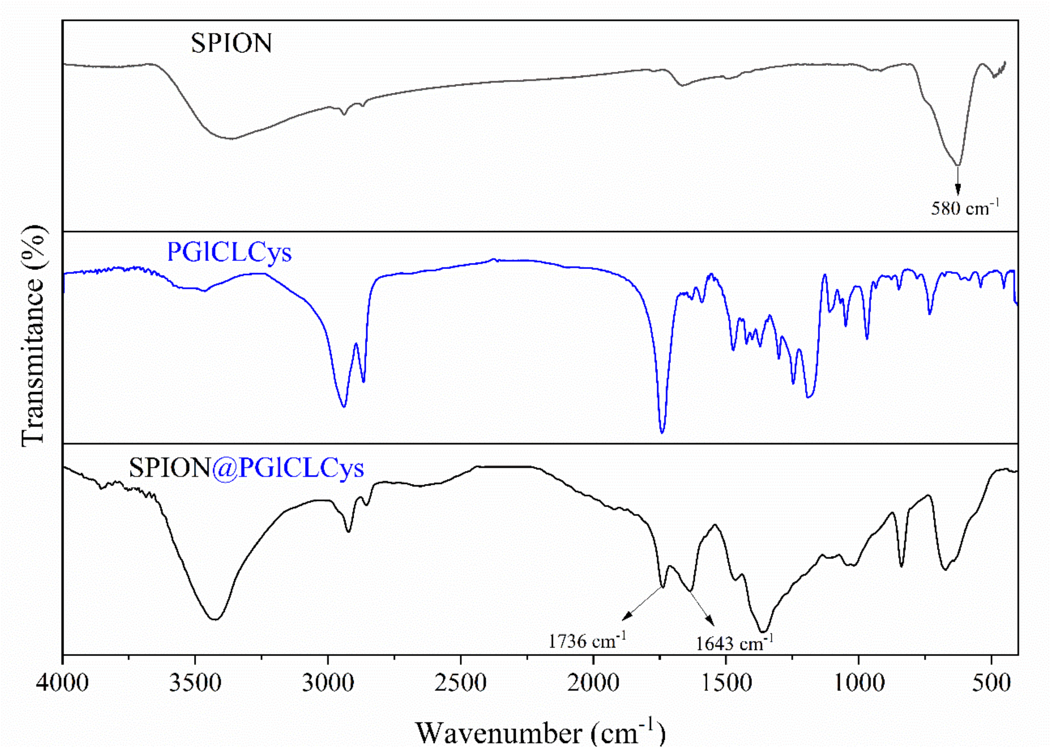

Figure S5. FT-IR spectra for SPIONs, and modified copolymer (PGlCLCys) and after coating of magnetic nanoparticles (SPION@PGlCLCys).

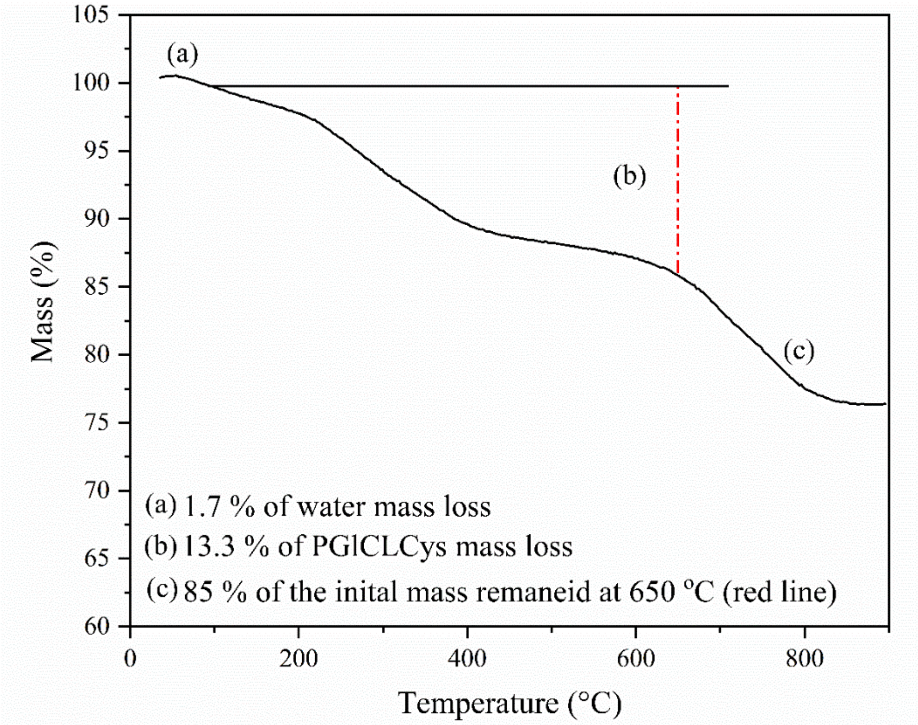

Figure S6. Thermogravimetric analysis of SPION@PGlCLCys.

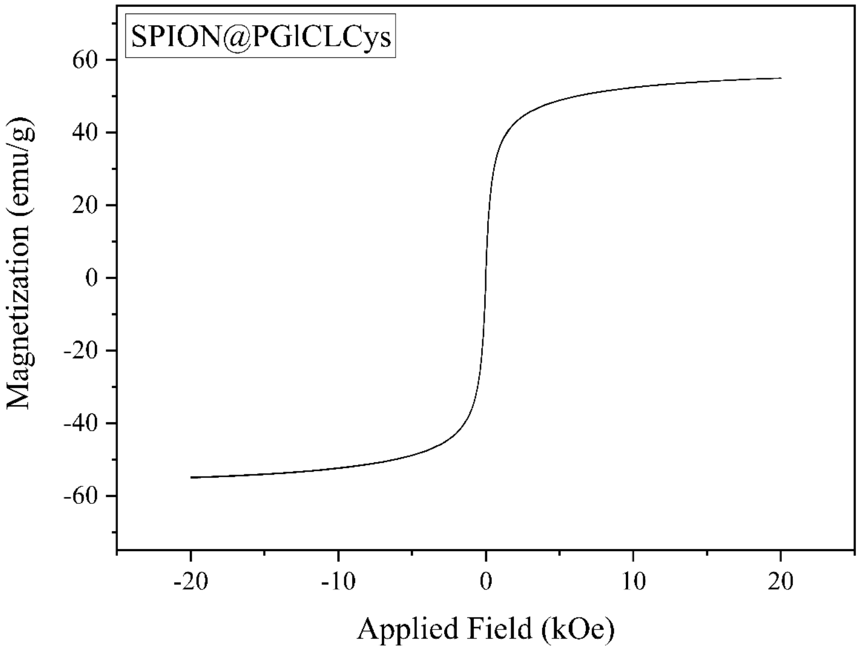

Figure S7. VSM analysis of SPION@PGlCLCys.

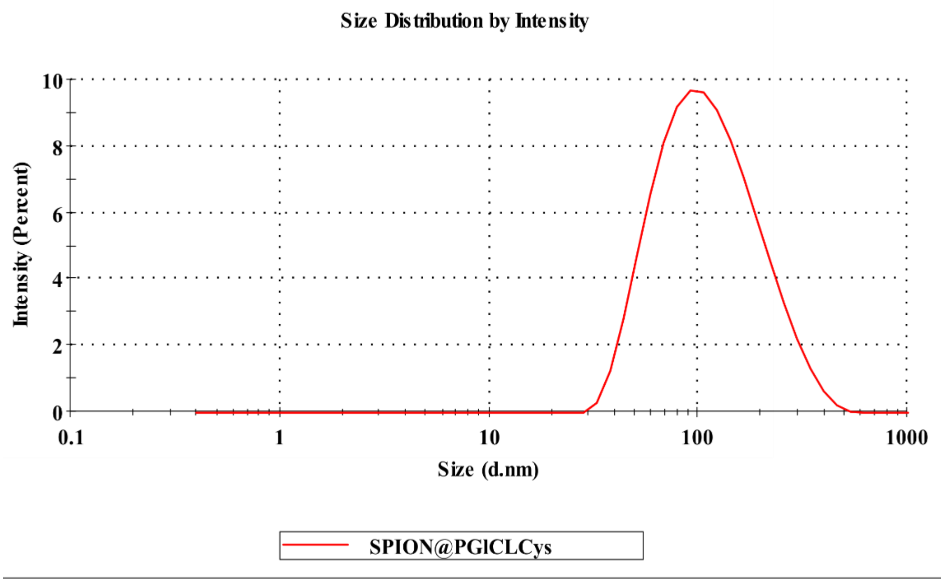

Figure S8. DLS analysis of SPION@PGlCLCys.

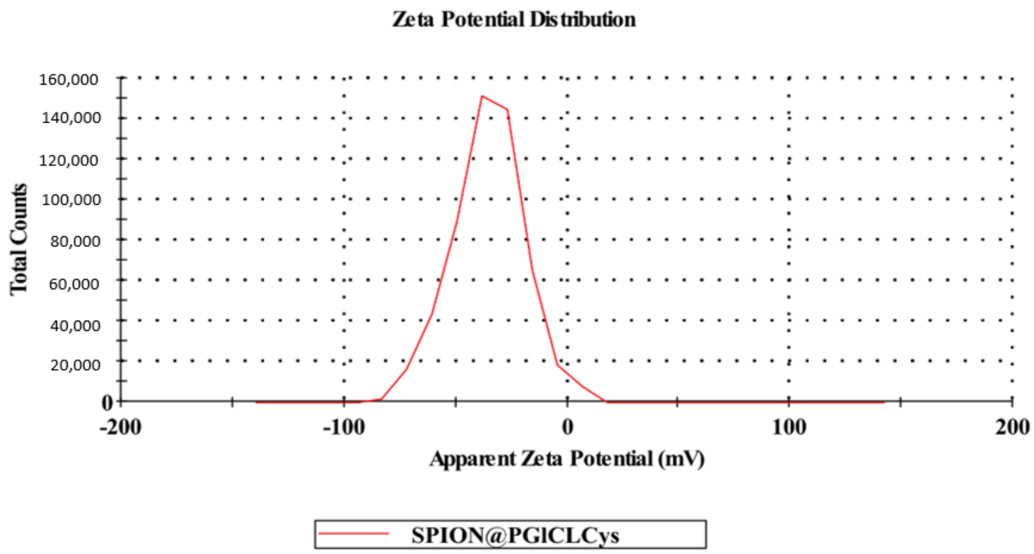

Figure S9. Zeta potential surface analysis of SPION@PGlCLCys (ζ= −35.4mV) dispersed in buffer solution (pH = 8.0).

A)

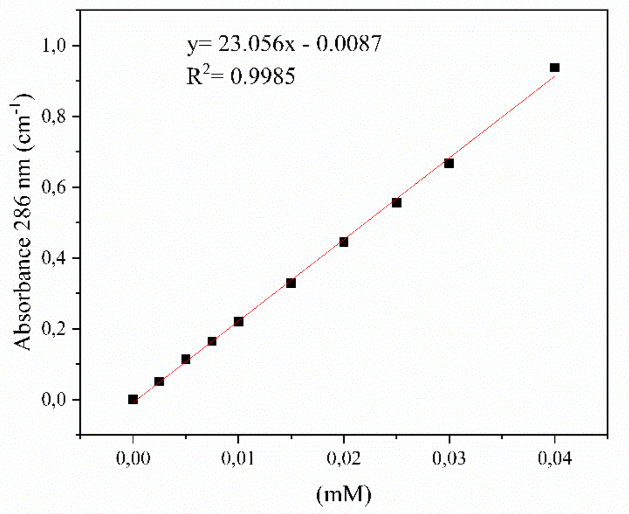

B)

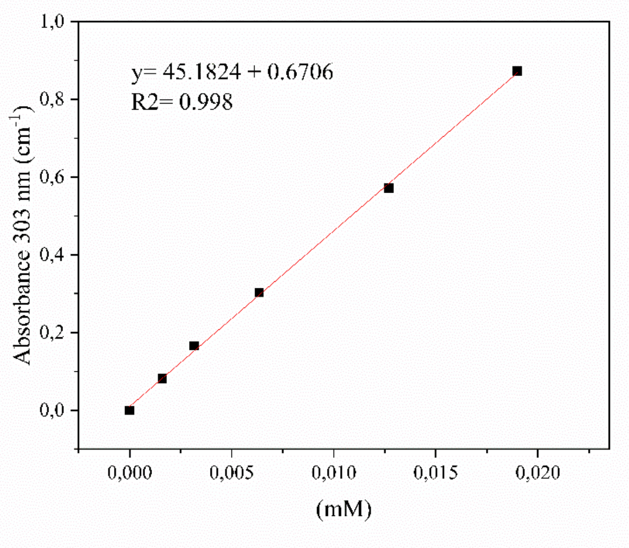

Figure S10. UV-vis calibration curve for: (**A**) folic acid (FA), and (**B**) methotrexate (MTX) in buffer solution (pH = 8.0).

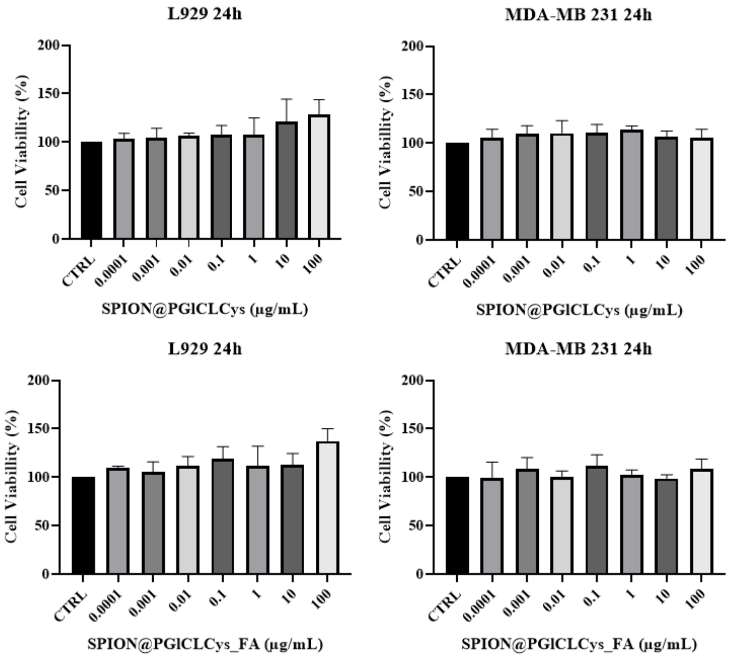

Figure S11: MTT assay of SPION@PGlCLCys and SPION@PGlCLCys_FA showing cells viability as a function of nanoparticles concentration (0.0001 to 100 µg·mL^{-1}) for 24 h. All SPIONs tested at different concentrations did not exert difference (ANOVA) in relations to the control group n=3.

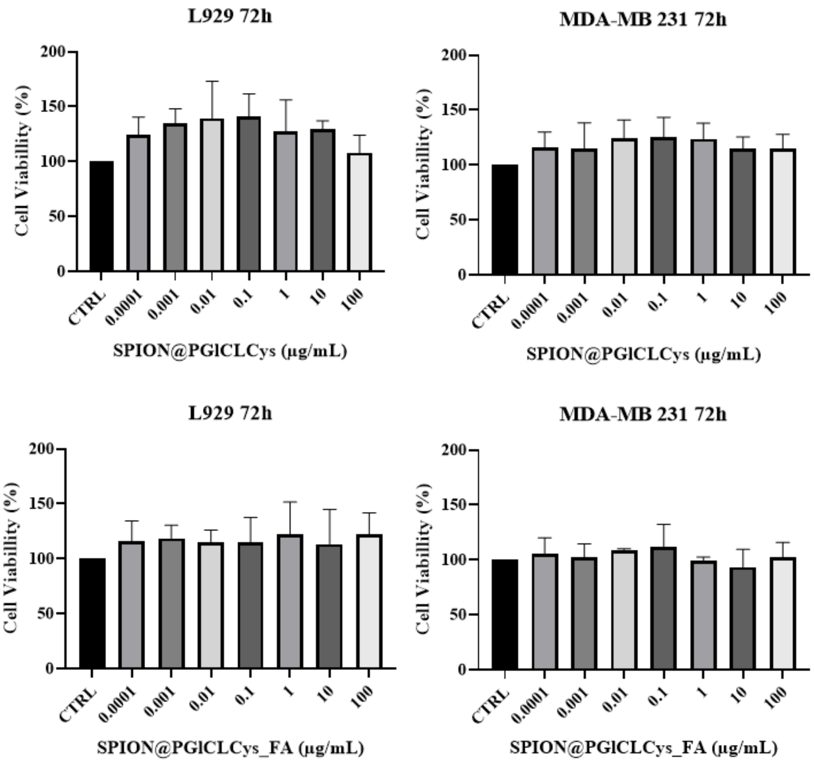

Figure S12. MTT assay of SPION@PGlCLCys and SPION@PGlCLCys_FA showing cells viability as a function of nanoparticles concentration (0.0001 to 100 µg·mL^{-1}) for 72 h. All SPIONs tested at different concentrations did not exert difference (ANOVA) in relations to the control group *n* = 3.

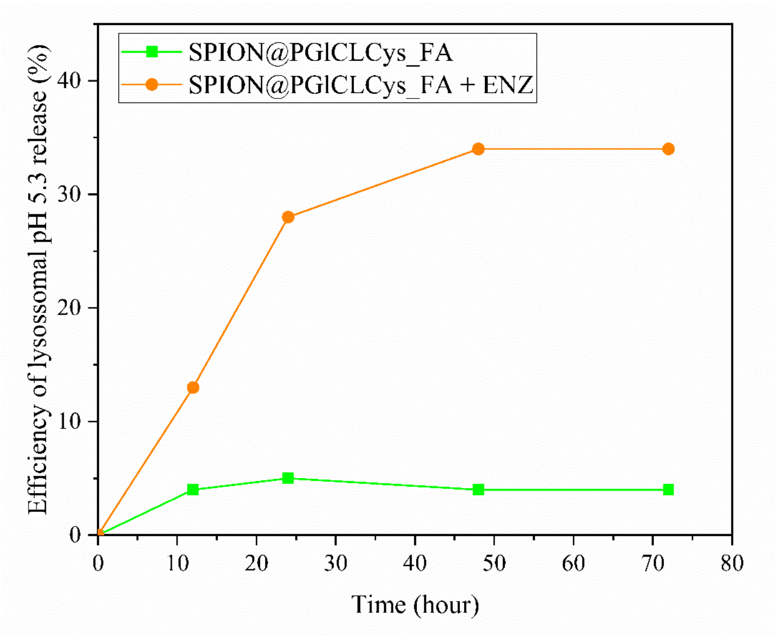

Figure S13. FA assay at lysosomal pH (pH 5.3).

References

1 V. Crescenzi, G. Manzini, G. Calzolari and C. Borri, *Eur. Polym. J.*, **1972**, *8*, 449–463.

2 M. E. Fleet, *Acta Crystallogr. Sect. B Struct. Crystallogr. Cryst. Chem.*, **1981**, *37*, 917–920.
